# Open cholecystectomy for all patients in the era of laparoscopic surgery – a prospective cohort study

**DOI:** 10.1186/1471-2482-6-5

**Published:** 2006-04-03

**Authors:** Jonas Leo, Goran Filipovic, Julia Krementsova, Rickard Norblad, Mattias Söderholm, Erik Nilsson

**Affiliations:** 1 Department of Surgery, Kirurgkliniken i Östergötland, Motala Hospital, Motala, Sweden; 2 Department of Surgery University Hospital, Umeå, Sweden

## Abstract

**Background:**

Open cholecystectomy through a small incision is an alternative to laparoscopic cholecystectomy.

**Methods:**

From 1 January 2002 through 31 December 2003, all operations upon the gallbladder in a district hospital with emergency admission and responsibility for surgical training were done as intended small-incision open cholecystectomy.

**Results:**

182 women and 90 men with a median age of 56 (interquartile range 45 to 68 years) underwent cholecystectomy for symptomatic gallbladder disease, 170 as elective and 102 as emergency cases. Trainee surgeons assisted by consultants or registrars having passed an examination for open cholecystectomy performed surgery in 194 cases (71%). The common bile duct was explored in 52 patients. Total postoperative morbidity was six percent. Median postoperative stay was one day and mean total (pre- and postoperative) hospital stay 3.1 days. 32 operations (12%) were done as day surgery procedures. Nationally in Sweden in 2002, mean total hospital stay was 4.4 days, and 13% of all cholecystectomies were performed on an outpatient basis.

**Conclusion:**

Open, small-incision cholecystectomy for all patients is compatible with short hospital stay, evidence-based gall-bladder surgery, and training of surgical residents.

## Background

Soon after its introduction, laparoscopic cholecystectomy was considered the method of choice for treatment of gallstone disease, and an early consensus conference concluded that it might confer economic advantages over open surgery[1]. At that time, little information was available concerning mini-laparotomy or small-incision, open cholecystectomy. Later single-blind, randomised controlled trials have indicated that convalescence differences between laparoscopic and small-incision surgery are small[2,3]. In previous reports from a controlled trial, no significant differences were observed between mini-laparotomy and laparoscopic cholecystectomy in terms of patients' opinion of general well-being, abdominal pain, and scarring one year after surgery[4,5]. Health-care costs are lower after mini-laparotomy cholecystectomy than after laparoscopic cholecystectomy [5-8].

Against this background it was appropriate to assess open, small-incision cholecystectomy as a treatment for all patients with gallstone disease in a district hospital with responsibility for surgical training. The assessment emphasised early surgery for patients with acute cholecystitis or mild pancreatitis, single-stage cholecystectomy and common bile duct clearance for patients with common bile duct stones, and surgical education of trainees.

## Methods

During the study period Motala District Hospital in Sweden was responsible for elective and emergency surgical care as well as for primary surgical training in cooperation with a nearby university hospital. According to the local training programme a trainee had to perform 50 open cholecystectomies under supervision and pass an examination on surgical skills and medical knowledge before undertaking an unsupervised cholecystectomy. Each cholecystectomy was prospectively recorded according to a protocol that involved patient characteristics, surgical details and intra-operative and postoperative complications, including re-operations. In spring 2004 all data were rechecked against hospital records. Postoperative complications were classified according to Clavien et al[9]. Numbers in brackets indicate severity of complication according to this scale.

Patients undergoing elective cholecystectomy were given verbal and written information concerning the operation, the expected hospital stay (including the possibility of ambulatory surgery), convalescence, and sick leave, which was recommended for one week. Those admitted through the emergency unit were transferred to a general surgical ward; from there they were placed on the operation list for cholecystectomy after diagnosis had been confirmed and preoperative measures begun. We tried to operate on patients with acute cholecystitis[10] in the acute or subacute phases of the disease, normally on the day after admission, and on patients with mild pancreatitis during the first hospital stay. An operation on a patient admitted through the emergency unit and placed on the operation list after confirmation of gallstone disease (acute cholecystitis, pancreatitis, jaundice/cholangitis, or severe biliary pain) was defined as an acute operation. Betametason 8 mg iv[11]; zolpidem 5–10 mg, ondansetron 4 mg, and paracetamol 1 g were given preoperatively p. o. and antibiotics (doxycycline or cefuroxime) were given to patients over 70 years of age as well as to patients with acute cholecystitis or signs of common bile duct stones. Thrombosis prophylaxis was administered as tinzaparin subcutaneously the evening before surgery or five hours after surgery.

The surgical technique described by Ledet and Seale was used[12,13]. Headlights and long, narrow retractors were routine and magnification glasses were recommended. A small cushion was placed under the caudal portion of the right thoracic cage in order to raise the gallbladder region. A transverse incision 4 to 8 cm in length, was placed over the right rectus muscle, approximately 5 cm below the xiphoid process. As a routine, the muscle was split longitudinally after transverse cutting of the anterior rectus sheath. The posterior rectus sheath was cut transversely. If distended, the gallbladder was emptied. The gallbladder was usually dissected from the fundus region and downwards. In case of severe inflammation with dissection difficulties, the gallbladder wall within the liver bed was left *in situ *and the mucosa cauterised. We tried to perform intra-operative cholangiography in all operations[14,15]. Suspicion of common bile duct stones with diameters exceeding 4 to 5 mm in combination with a wide common bile duct (10 mm) was considered an indication for exploration of the common bile duct. Stone extraction through the cystic duct was tried initially; if it was unsuccessful, the common bile duct was opened longitudinally to allow stone removal. In case of smooth bile duct clearance primary closure was performed[16], otherwise a T-tube was placed in the common bile duct. Ringer's solution was used for washing the abdominal cavity before wound closure[17]. Local anaesthesia, 20 – 40 ml of bupivacin 0.25% mg, was infiltrated in the wound at the end of surgery. Early mobilisation was encouraged. Paracetamol was recommended as routine pain medication for five days, if necessary supplemented by diclofenac. Those who were treated on an outpatient basis were contacted by telephone on the first postoperative day.

As minilaparotomy cholecystectomy and laparoscopic cholecystectomy were compatible with routine surgical practice, ethics approval was not sought.

## Results

From 1 January 2002 through 31 December 2003, 182 women and 90 men, age between 12 and 91 years old (median 56), underwent cholecystectomy. 170 cholecystectomies were done as elective procedures and 102 as acute operations. Ultrasonography confirmed the gallstone diagnosis for 268 patients, computerized tomography in three cases, and endoscopic retrograde cholangiography in one case.

Gender, American Society of Anaesthesiologists' (ASA) score, age, and BMI of patients are shown in Table 1. Trainee surgeons assisted by consultants or registrars having passed the examination for cholecystectomy performed surgery in 194 cases (71%), and consultants did the procedure in 78 cases (29%). Intra-operative cholangiography was performed in 261 cases (96%). The common bile duct was explored in 52 patients (19%). In three cases this was done through the cystic duct. Common bile duct stones were extracted or gently pushed down to the duodenum. Choledochoscopy was undertaken in 38 cases.

Length of skin incision, operation time, and postoperative hospital stay are given in Table 2 for elective and acute cases separately. 32 patients (12%) were operated on as day-surgery cases and 111 patients spent one night in hospital. Median and mean postoperative stay for all patients was one day and 2.1 days, respectively. Postoperative stay was 1.8 days for 182 patients with wound incision shorter than 8 cm compared to 3.0 days for 87 patients with incision 8 cm or longer (data missing for 3 patients). Total hospital stay, preoperative stay included, amounted to 3.1 days (mean) for all patients.

Morbidity of all patients was six percent. Altogether five patients were re-operated. One 81 year-old woman admitted with acute cholecystitis and perforated gallbladder died in multiorgan failure after re-operation for bleeding (IV). Another patient with severe acute cholecystitis, who was re-operated because of bleeding from the liver bed made an uneventful recovery (IIb). One 76 year-old man, admitted with jaundice and cholangitis, had previously undergone subtotal gastrectomy because of severe pancreatitis. Bile leak was seen after cholecystectomy, and at re-operation a carcinoma in the distal common bile duct was found, unidentified on repeated computerized tomographies before the diagnostic laparotomy (IIb). A palliative operation with bile diversion was performed after referral to a hepatobiliary centre. Leakage from the cystic duct required open drainage, endoscopic cholangiography and temporary endoprosthesis (IIb) in one patient. Finally, one patient underwent open drainage for deep infection (IIb).

Postoperative complications treated without re-operation were noted in 12 patients. In one patient calculi in the common bile duct stones had to be removed with sphincterotomy and temporary endoprosthesis (IIb); another patient was erroneously administered low molecular weight heparin intra-operatively and developed multiple intra-abdominal haematomas, which resolved spontaneously (II b). Wound infections were identified in 10 patients (grade I in eight patients and grade IIa in two patients). Patients with wound infection were older than those without wound infection, mean 67 versus 54 years, but they did not differ significantly from patients without wound infection with respect to emergency indication (30% versus 38%) or BMI (mean 27.3 versus 28.2).

## Discussion

In the present cohort study of cholecystectomy with intended small-incision cholecystectomy for all patients (and no laparoscopic cholecystectomy), one-third of all operations were acute procedures. Exploration of the common bile duct was done in 52 of 272 patients. Median postoperative hospital stay was one day, total mean hospital stay (pre-and postoperative stay) 3.1 days, and 12% of all procedures were done on an outpatient basis.

The strength of this report is the inclusion of all cholecystectomies performed in one unit with emergency admission during a two-year period. Mini-laparotomy cholecystectomy is usually defined as open cholecystectomy through an incision of 4 to 7 cm[8,13] or less than 6 cm[18]. In this prospective and consecutive series, median length of incision was 7 cm for elective operations and 8 cm for acute operations. This demonstrates that surgical training and safety were prioritised in the present study, and it also indicates possibilities of further improvements in day-case rate and convalescence.

Operation time included intra-operative cholangiography in 96% of the cases, common bile duct exploration in 19%, and training of surgical residents in 71% of all operations. The complication rate in this series was six percent. Eight of 17 complications were wound infections of minor clinical importance (Clavien grade I). In previous randomised controlled trials comparing mini-laparotomy cholecystectomy and laparoscopic cholecystectomy complication rates between 3 and 20% have been observed without significant difference between the two techniques[2,3,19-22]. Total hospital stay in our study was 3.1 days (mean) with 12% of all procedures done as day-cases. These figures compare favourably with national statistics for gallbladder surgery. In 2002, 12,357 cholecystectomies were done in Sweden, and 9,836 of these were completed laparoscopically[23]. The day surgery rate for *laparoscopic *cholecystectomy was 17%, or 13% of all cholecystectomies. The mean hospital stay was 2.7 days for laparoscopic cholecystectomy, 8.8 days for open cholecystectomy, and 4.4 days for all cholecystectomies[24], i.e. approximately one day longer than in our study.

We utilised no screening program for common bile duct stones and had to rely on endoscopic stone removal for two patients postoperatively. Frequent use of intra-operative common bile duct exploration minimised the use of postoperative endoscopic sphincterotomy with its inherent risk of rare but serious complications[25]. Randomised controlled trials of open[26,27]. and laparoscopic[28,29] cholecystectomy have shown that single – stage treatment of common bile duct stones, i.e. cholecystectomy and common bile duct clearance during the same operation, is preferable compared to bile duct clearance before or after cholecystectomy. Early surgery is the optimal treatment for acute cholecystitis (within seven days of the onset of illness)[30], and in mild gallstone pancreatitis surgery should be considered within two to four weeks[31]. Surgical education should therefore prepare the trainee for emergency or urgent gallbladder surgery.

The main advantage of using small-incision open cholecystectomy for all patients is its general applicability and elimination of double learning curves. Nationwide studies have shown that after the introduction of laparoscopic cholecystectomy 20 to 30% of all gallbladder operations are completed openly, and that patients thus treated are older and have more co-morbidities than patients undergoing laparoscopic cholecystectomy [32-34]. From 1995 through 1999, 82% of Swedish patients over the age 70 treated for acute gallstone disease and 43% of those treated for chronic gallstone disease had an open operation[35]. The limited exposure to open biliary surgery creates a dilemma for training of residents[36,37]. The surgical community has to develop strategies to meet the growth of workload accompanying the increasing age of populations in the western world[38]. The present cohort study indicates that small-incision open cholecystectomy is an attractive alternative for elderly patients, with their high incidence of acute cholecystitis and common bile duct stones[39]. We agree with Syrakos et al[8] that commissioners of health care should question whether laparoscopic gallbladder surgery gives value for the cost. Further cost-utility studies comparing mini-laparotomy cholecystectomy and laparoscopic cholecystectomy are necessary, ideally performed as expertise based randomised controlled trials[40]. As pointed out earlier, register studies with their inherent difficulties in controlling for patient characteristics are unlikely to answer questions concerning relative merits cholecystectomy techiques[35,41].

## Conclusion

Open cholecystectomy, with intended mini-laparotomy cholecystectomy, is compatible with short hospital stay, evidence-based gall-bladder surgery, and training of surgical residents.

## Competing interests

The author(s) declare that they have no competing interests.

## Authors' contributions

E N initiated the study; all authors contributed to the surgery performed, to the prospective collection of data after each operation and after hospital discharge of the patient. J L and J K scrutinised the prospectively collected data. J L and E N wrote the manuscript which has been seen and approved by all authors.

## Pre-publication history

The pre-publication history for this paper can be accessed here:



## Figures and Tables

**Table 1:** Characteristics of patients

**Gender, ASA – score**				
	Men	Women	ASA I & II	ASA III & IV

Acute	39	63	90	12
Elective	51	119	152	18

**Age, BMI**				

		Age, years	BMI	

Acute	25 percentile	41	22	
	Median	61	26	
	75 percentile	76	28	
Elective	25 percentile	39	25	
	Median	53	27	
	75 percentile	66	31	

**Table 2:** Skin incision, operation time, and postoperative hospital stay

		Skin incision (cm)	Operation time (min)	Postoperative hospital stay (days)
Acute	25 percentile	4	85	1
	Median	8	98	2
	75 percentile	20	149	4
Elective	25 percentile	4	85	1
	Median	7	104	1
	75 percentile	15	127	1
